# Abstract social categories facilitate access to socially skewed words

**DOI:** 10.1371/journal.pone.0210793

**Published:** 2019-02-04

**Authors:** Jennifer Hay, Abby Walker, Kauyumari Sanchez, Kirsty Thompson

**Affiliations:** 1 Department of Linguistics, University of Canterbury, Christchurch, New Zealand; 2 New Zealand Institute of Language, Brain & Behaviour, University of Canterbury, Christchurch, New Zealand; 3 Department of English, Virginia Polytechnic and State University, Blacksburg, VA, United States of America; 4 Department of Psychology, Humboldt State University, Arcata, CA, United States of America; University of Hull, UNITED KINGDOM

## Abstract

Recent work has shown that listeners process words faster if said by a member of the group that typically uses the word. This paper further explores how the social distributions of words affect lexical access by exploring whether access is facilitated by invoking more abstract social categories. We conduct four experiments, all of which combine an Implicit Association Task with a Lexical Decision Task. Participants sorted real and nonsense words while at the same time sorting older and younger faces (exp. 1), male and female faces (exp. 2), stereotypically male and female objects (exp. 3), and framed and unframed objects, which were always stereotypically male or female (exp. 4). Across the experiments, lexical decision to socially skewed words is facilitated when the socially congruent category is sorted with the same hand. This suggests that the lexicon contains social detail from which individuals make social abstractions that can influence lexical access.

## Introduction

The distribution of word usage across different speaker groups is not even. Some groups may talk about certain topics more than others or use different words to talk about the same topics. This leads to certain words being used more by some types of speakers than others. For example, the word *children* is more likely to be said by women than men [1]. Therefore each word has a unique social distribution in which it is encountered, or–in the words of Bakhtin–“[e]ach word tastes of the context and contexts in which it has lived its socially charged life” [2](p.293). Recent results suggest that speakers and listeners are sensitive to the differing social distributions of individual words. For example, in speech perception, words are accessed faster when produced in experience-congruent voices [3, 4], and in speech production, these words are more likely to be produced with experience-congruent phonetic variants [5, 6].

Such findings have been used to argue for exemplar accounts of language and memory, in which cognitive representations of words consist of accumulated distributions of past experiences (exemplars) of the word, complete with acoustic and contextual detail [7–10]. In such models, a person’s mental representation of the word *bead*, for example, would be a distribution of remembered exemplars of previously experienced “bead” utterances. Speech production involves selecting an exemplar from this distribution, or the average of many exemplars, to emulate [10], and speech perception involves matching the incoming signal to existing exemplars based on acoustic and contextual similarity [11]. Such models are supported by specificity effects. For example, German et al. [12] found that North American participants trained on the speech of a Glaswegian speaker were best at producing Glaswegian variants in the particular words used at training (see also [13–15], and others). This is difficult to explain without arguing that the representations of the specific words heard in the Glaswegian accent were individually affected by the training experience.

In a similar way, results showing listener and speaker sensitivity to word distributions can also be explained by word-specific, phonetically-detailed memories. If a listener has encountered a word most often from older speakers, then hearing that word in an older voice should facilitate lexical access due to a phonetic match between the voice of the stimuli and the voices that dominate the word’s cognitive representation. Such an account can predict congruency effects between word experience and stimulus voice, as shown in Walker and Hay [3]. Similarly, in production, a word used more often by older speakers will be stored with a higher proportion of ‘older’ productions, and thus be more likely to be produced with a variant that is associated with older speakers, as shown by Hay & Foulkes [5].

Exemplar models contrast with models that posit that words are represented by abstract, phonological entries [16]. For example, in terms of pronunciation, a person’s mental representation of the word *bead* would be /b/ + /i/ + /d/. Speech production involves articulating these phonemes in order, and speech perception involves normalizing or filtering variation in the signal to arrive at the correct phonological parse. Such models are supported by *generalization* effects, where someone applies learning or associations beyond what they have directly experienced. For example, in the previously mentioned German et al. [12] paper, while participants produce Glaswegian variants most often with words they had directly experienced in the Glaswegian accent, they critically also showed generalization to words *not* used in training. Such generalization has been found to occur across allophonic boundaries [17, 18], across speakers [19], and across features [20, 21].

Effective models of language processing then must be able to account for both specificity and generalization, and many researchers argue for hybrid models, where word representations contain *both* specific, detailed phonetic memories of words (exemplars) and also include higher level abstractions (i.e., the phoneme category [i]), both of which serve to impact speech production and perception [12, 22–24]. Thus, for any particular word, we have a phonetically detailed distribution shaped by past experience as well as stored associations with the relevant higher-level categories: the word *bead* has a detailed distribution which is word-specific, and is also associated with the more abstract categories of /b/, /i/, and /d/. In such accounts, encountering a new entry of “bead” would update the representation of both the specific word, and also the associated phonemic categories.

The motivation for hybrid models has been the clear impact of both detailed acoustic memories and abstract phonological categories in speech processing; that is, the argument for hybrid models has been based on *sounds*. But in exemplar models, memories are not simply rich in acoustic detail, but also rich in other sensory and contextual details: physical sensations [25], physical location [26], visual presentation [27–29], and critically for this paper, social information about the speaker (see Foulkes & Docherty [30] for a review). It is therefore likely that people also generalize from statistical associations between a word and particular types of speakers to associations between that word and more abstracted *social* categories. Indeed, a number of recent models of lexical representation and perception very explicitly assume that our experiences of words are simultaneously interpreted for both linguistic and social meaning–activating, for example, both phonological categories, and social categories. For example, Munson [31] provides a schematic of a rich hybrid exemplar theory, in which the incoming signal is simultaneously indexed to multiple levels of representation–both social and phonological. And Sumner et al.’s “dual route approach to speech perception”, assumes that “learned acoustic patterns are mapped simultaneously to linguistic representations and to social representations” [32](p.1).

While often assumed, the role of multiple levels of social representation for words has not been very well explored, though there are many reasons to believe such a relationship exists. Just as we organize continuous, phonetic information into categorical, phonological units [33, 34], there is evidence for similar, categorical perception of social information [35, 36]. Moreover, attributes of a social group generalize beyond the individuals in a group to *objects* associated with the group. For example, Lemm, Dabady, & Banaji [37] conducted a priming experiment to test whether images that only connotatively referenced gender (i.e., oven mitts and baseball mitts) could prime FEMALENESS and MALENESS similar to the way that words and images with denotative gender (images of faces, words like *congresswoman*) do. They found that images that connotatively referenced gender did indeed prime notions of gender (though to a lesser degree than denotative gender items), arguing that

…masculinity and femininity can be activated even by stimuli that are not exclusively male or female (e.g., sports cars or frilly lampshades, or job titles such as pilot or nanny). Gender concepts pervade ordinary objects and words; even stimuli that do not have an intrinsic gender can bring gender concepts readily to mind upon the briefest exposure. (p. 236)

Their finding is consistent with other work that indicates that stereotypes abstract beyond members of that group to objects or words related to the stereotype [38–40].

Therefore, just as we see generalization from experienced exemplars based on phonemic labels, we should also see evidence of generalization based on social labels. There is already some indirect evidence for such social abstractions at the *sound* level (as opposed to the *word* level). Hay & Drager [41] used stuffed toys associated with New Zealand or Australia and were able to prime New Zealand and Australian dialects respectively in a speech perception task. It is unlikely that participants’ experiences of Australian and New Zealand English were always accompanied with stuffed toys, so this effect appears to stem from the shared association of both the toys, and certain phonetic features, with the labels AUSTRALIAN or NEW ZEALAND. Szakay, Babel, & King [42] demonstrate that if words in New Zealand English are produced with phonetic variants consistent with Māori English, their Māori translation equivalent is primed more than if they were produced with more Pākehā (non-Māori) variants.They argue that this is because the phonetic variant and the language (Te Reo) Māori are both associated with the social category of MĀORI.

In the current study we set out to test whether people generalize from experiences with specific speakers at the *word level* to social information about those speakers and to social categories more generally. For example, if certain words are experienced more in female voices compared to male voices, this should build a relationship not simply between the acoustic gender of the voice and the word (*specificity*, see [3]), but more generally between the word and other things that share the label FEMALE (*generalization*). That is, we should see a relationship between female gendered words and female gendered objects, like a handbag, even though the words and objects did not necessarily co-occur in people’s experiences.

We want to emphasize at this point that terms like “gendered words” in this paper refers to words that are relatively over-represented in corpora of female vs. male speech, not necessarily stereotypically or denotatively gendered words. Our previous work [3] suggests that while there is a correlation between these corpus-based ratios and participants’ conscious awareness of usage biases, the corpus counts predict processing behavior where ratings do not (though see [4]). In fact, sometimes usage-gender and denotative or connotative gender might actually conflict, such as in words such as *husband*, which is used more by women than men, but denotes a man.

We ran four experiments to test the primary hypothesis that participants abstract from speaker-specific associations of a word to category general associations. All of the experiments use a combination of the Implicit Association Task (IAT) and Lexical Decision Tasks (see 1.1). The first two experiments build on Walker & Hay [3], which examined congruency effects between word age/gender in a lexical decision task. Our experiments test whether participants show evidence of an implicit association between age-skewed words and OLD/YOUNG (experiment 1), and between gender-skewed words and MALE/FEMALE (experiment 2), using faces in the sorting task. The third experiment takes a more abstract step, again examining whether participants have an implicit association between gendered words and MALE/FEMALE, but using gendered objects instead of faces in the sorting task. The fourth experiment also tests the relationship between gendered objects and words, but without explicitly mentioning gender in the task.

### Investigating lexical access using the Implicit Association Task

The Implicit Association Task (IAT) [43] aims to measure the associative strength between different concepts/objects/groups. In the IAT, participants need to pair two different dimensions, and the ease with which they do so is taken as evidence of the associative strength of these dimensions. For example, Greenwald and colleagues used this procedure to test implicit attitudes toward ethnicity. Participants are asked, across separate tasks, to sort names of objects as PLEASANT or UNPLEASANT (e.g. *flower*, *insect*), or to sort proper names as BLACK and WHITE (e.g. *Latonya*, *Meredith*). The critical portion of the experiment involves completing both tasks within a single block. If participants are faster and more accurate at the task when BLACK and UNPLEASANT are responded to with the same hand than when BLACK and PLEASANT are paired, this is understood to reveal the participants’ implicit, negative attitude or association with black people (the sorting task is easier because the participant implicitly sees BLACK and UNPLEASANT as being similar categories). Across many studies, researchers have used variants of this task to attempt to tease out implicitly held associations, most commonly testing implicitly held attitudes of their participants.

Social psychologists often use words as stimuli to invoke the social concepts under comparison, and linguists have used IATs to measure underlying social prejudices of speakers [44–46]; however, little research has directly used the IAT to investigate linguistic processing. An exception is Campbell-Kibler [47, 48], who has used the IAT to investigate sociolinguistic meaning, showing that participants implicitly associate sociophonetic variants with various social dimensions (region, profession).

In the current study, we combine the IAT with a visual Lexical Decision Task. In Lexical Decision Tasks, participants are presented with a word and asked to decide, as quickly as possible, whether the word is a real word or not. Their accuracy and response times are thought to reflect ease of lexical access, and a common finding is that people recognize high frequency words as real words faster than low frequency words [49, 50]. Of the most relevance to our study, researchers have shown that access to words that are more often used by older speakers [3, 4] or women (see Section 3.1.1) is facilitated when the word is presented in an older or a female voice respectively.

In our combined IAT and Lexical Decision Task, participants simultaneously sort words into real words or nonsense words, and images/text into categories (in our study, age or gender categories). At a given block in the experiment, participants use the same hand to sort real words and, for example, female faces. Historically, when psychologists have combined these tasks, they have usually been exploring the mechanisms behind IAT tasks [51, 52]. For example, Rothermund & Wentura [53] use a non-word/word sorting task in place of a positive/negative sorting task in an old-young IAT. They show that they get NON-WORD+OLD facilitation much akin to the well attested UNPLEASANT+OLD effect [54], and use this finding to argue that the symmetrical *salience* of paired categories might be driving facilitation in the IAT, more so than shared semantic or evaluative associations. That is, the reason participants do better with both the NON-WORD+OLD and the UNPLEASANT+OLD pairing is because in both cases, the salient category old is paired with the salient categories of non-words, or negative things. In contrast, real words, positive things, and youth share being non-salient, or unmarked. Greenwald et al. [55] respond that regardless of what exactly is being measured, “the implications for construct validity of IAT measures are the same” (p. 425), though debates continue [56].

Our study differs from these previous studies that have used a Lexical Decision Task as part of the IAT in that we analyze the data like a Lexical Decision Task: we are interested in the differences in responses to different real words (i.e., a within-category difference), which we take to reflect speed of lexical access, and we investigate this by looking at accuracy rates and response times in real words trials only, as a function of what other category they are paired with. Critically, our aim is not to investigate the associative strength between the categories of REAL WORD and FEMALE, etc., though this is a byproduct of our design. Rather, we want to test the overarching association between individual stimuli (words) and a social category (e.g. OLD/YOUNG or FEMALE/MALE) using primary (faces) and secondary (objects) associations of these social categories.

We predict that participants will be faster at recognizing older/female words when the hand they use to sort older/female faces is the same hand they use to sort real words, reflecting an implicit association between the word and older/female speakers (and vice versa for young or male words). Moreover, we expect that this association will hold even when the sorting task involves gendered objects, not faces (experiment 3) and when we remove any mention of “gender” from the task (experiment 4). Our predictions are based on two assumptions: first, that people have access to the statistical associations between certain words and the populations of speakers who use them [4, 5]; second, that beyond this, they generalize to make associations between words and abstract social categories.

## Experiment 1: Older and younger words, older and younger faces

This experiment tests for an implicit association between word age and the categories OLD and YOUNG. In this experiment, participants engaged in a combined IAT and Lexical Decision Task where they sorted words and non-words while also sorting old and young faces. The words came from Walker & Hay [3] and were words that were skewed in their usage across speakers of different ages, such that older speakers were more likely to use some of these words than younger speakers, and vice versa. We predict that participants will be faster at accessing real, older words if they are using the same hand to sort real words and older faces. If we find a significant facilitation effect of face and word congruence, this would provide support that words used more by older or younger speakers are associated with older and younger people respectively.

### Methodology

#### Stimuli

The stimuli in this experiment consisted of photographs of older and younger faces, and single, orthographically presented words. The 80 words in this study consisted of 40 real words and 40 non-words, and were a subset of the words used by Walker & Hay [3]. The real words were sourced from two corpora from the Origins of New Zealand English (ONZE) archives [57], a growing repository of recorded and transcribed interviews of native speakers of New Zealand English, housed at the University of Canterbury.

Conceptually, we use this corpus to represent the experienced speech of our participants, a crude but common tactic used to explore frequency effects [3,10]. It is crude for two reasons. First, experience is individual: no two people will have experienced the same samples of speech in their lifetime, and certainly no one would have received only and all of the speech represented in ONZE. Second, there is ample evidence that not all linguistic experience is attended to or encoded in memory in the same way [see 32 for a review]. Our use of a corpus then represents a methodological necessity rather than a theoretical claim about what sort of speech is committed or not committed to memory. Therefore, we proceed as if the corpus reflects our participants’ experiences, and as if all of these experiences are equally well stored in memory.

To choose age-skewed words, we compared word frequencies in the Intermediate Archives (IA) (> 515,000 words from ~90 speakers born between 1890–1930) relative to the Canterbury Corpus (CC) (> 815,000 words from ~400 speakers born between 1930–1984 at the time we extracted data). The 40 real words were selected by comparing the relative frequency of words in the IA and CC, and noting when words were overrepresented in one corpus relative to the other. 20 words that skewed old in their usage were selected, which were between 30:1 (*fireworks*, *confectionery*, *frighten*, *idle*, *mittens*, *pencils*, *willow*) and 7:1 (*fried*) times more common in the IA than the CC, and had an in-IA corpus frequency range of 10 to 64 ppm (parts per million). The 20 young words ranged from being around 22:1 (*bitten*, *chemistry*, *depressing*, *impressive*, *intellectual*, *physics*, *spirits*) to 5:1 (*nicest*, *environment*) times more common in the CC versus IA, and had a within-CC frequency range of around 9–121 ppm in the CC corpus. There was no significant difference between the frequency of old words in the IA (*mean* = 24.72ppm, std.dev = 15.75), or young words in the CC (*mean* = 32.28ppm, std.dev = 31.94) in a Wilcoxon rank sum test (W = 190.5, p = 0.8075).

Forty non-words were created from the real words, maintaining stress pattern, syllable structure, and orthographic length, and with legal English phonotactics (see Appendix A for the list of real and non-words). A task completed at the end of the experimental session asked participants to rate the real words on a 6-point scale of age usage, from 1, meaning that this words is “much more likely to be used by younger speakers” to 6, meaning this words is “much more likely to be used by older speakers”. Old words received an average rating of 4.04 (sd = .76), and young words received an average rating of 3.66 (sd = .58). A Wilcoxon rank sum test did not find this difference in overt perceptions of word use depending on age to be significant (W = 268.5, p = 0.07), suggesting that listeners are not consciously aware of the patterns of the skewed usage of these words by older/younger speakers.

The photographs in this experiment depicted young and old faces. To produce the photos, candidate subjects were recruited via personal contacts. Photographs of their faces were taken in front of a plain white background while they directly faced the camera with a neutral facial expression and both eyes open. To avoid recognition of the identity of the face, which may alter the intended results of the experiment [58], novel faces were created using the software Abrosoft FantaMorph [59]. Each original face was morphed with another face of his or her own sex to create a unique face. In total, 20 unique photographs were created depicting unique faces (10 old, 10 young). There were five photos of each sex for each age classification. All photos appeared to be natural to the authors (examples available at https://github.com/jenniferhay/hayetal-plos2019/supplementaryfigures.pdf).

#### Participants

Forty-two participants (32 female, 10 male) were recruited at the University of Canterbury and compensated with a $10 voucher for their time. Participants were aged between 18 and 56 (median year of birth = 1991). All participants were native or near-native speakers of New Zealand English (moved to New Zealand before the age of seven).

#### Procedure

Participants individually engaged with the experiment in a quiet room over a single session. The fourth author (a young, female, native speaker of NZE) ran all participants. The experiment was programmed in E-Prime 2.0 software (Psychology Software Tools, Pittsburgh, PA) and was run on a dual operating system Macintosh computer while in the Windows operating system. Participants were instructed that they were in a reaction time experiment and were encouraged to respond as quickly and accurately as possible.

There were seven blocks in the experiment. In the first block, participants sorted the 20 morphed photographs of faces by the age of the face. For half of the subjects, the word “Older” appeared on the top left side of the computer screen and the word “Younger” appeared on the top right side, which was reversed for the other half of subjects. The randomly selected target stimulus (i.e. photo of a face) appeared in the center of the screen. Participants were asked to quickly categorize the photo with the left (q) key if it was an older face or the right (p) key if it was a younger face. For trials in this and all subsequent blocks in which participants sorted on a single dimension, participants were allowed 1500ms to categorize each photo before they were prompted to “respond faster”. Similarly, if the participant responded correctly the next trial would commence. An incorrect response would prompt a red “X” to appear at the bottom center of the screen, with the next trial commencing only when the participant selected the correct response for the trial.

In the second block, participants sorted the words by whether they were real or nonsense words (i.e., a Lexical Decision Task). For all subjects the label “Real” appeared on the top left side of the computer screen and the word “Not Real” appeared on the top right side. The randomly selected target stimulus (i.e. word or non-word) appeared in the center of the screen. Participants were asked to quickly categorize the word or non-word with the left (q) key if it was a “Real” word or the right (p) key if it was a word that was “Not Real”.

The format for the third and fourth blocks was identical, with the short third block serving as practice for the fourth block. Here, the categories from blocks one and two were combined (i.e., an IAT), so that participants sorted REAL words with their left hand, NOT REAL words with their right hand, and the photos into OLDER or YOUNGER with the hands they had used for the first stage of the experiment. The third block contained four trials, one per each type of stimulus label. During all practice trials, participants were given an infinite amount of time to respond. The fourth block contained 160 randomized trials: the 80 words and the four presentations each of the 20 photos. To allow for the increased difficulty of sorting on two dimensions, participants were given 2000ms in this block (and in block 7), before they were prompted to respond faster.

The fifth block was the same as the first block, but with the category labels switched, so that if a participant originally categorized a face as OLDER with their left hand, they now categorized it with their right. The sixth and seventh blocks were identical to blocks three and four, respectively, but with the new age-categorization labels learnt in block five. This experiment, and all subsequent experiments reported in this paper, were reviewed and approved by the Human Ethics Committee of the University of Canterbury.

#### Analysis

All data were analyzed using R [60] and the R packages lme4 [61] and languageR [62, 63]. Mixed effects models were fit by hand, using model comparison to select the best-fit model. The dependent variables consisted of accuracy (logistic regression) and log reaction time (linear regression), run in separate analyses. The random intercepts in the analyses were always Subject and Word [64, 65]. The tested fixed effects consisted of Participant Age (a median split: born in 1991 or later = younger participants; born before 1991 = older), Word Age (of stimulus as selected via the ONZE Corpus: older or younger–see [3], Pairing (if the age of the photo and the REAL label is paired with the same hand: OLDER-REAL, YOUNGER-REAL), Handedness (of the participants, right or left), Block, and Trial number (scaled and centred–within block). Model fitting began with all listed fixed effects, and all three-way interactions between Trial, Participant Age, Word Age, and Pairing. Non-significant interactions and fixed effects were iteratively dropped from the model, based on anova comparison. In all models, random slopes were included for Word Age and Pairing (and their interaction) on the participant intercept.

Reaction time cut-offs were used to filter the data. The lower bound cut-off was set to responses faster than 250ms, while the upper bound cut-off was set to responses slower than two standard deviations above the mean within a participant’s data. In experiment 1, 6.7% of the data were removed with this method.

### Results

#### Accuracy

The overall accuracy rate was 94.1%. The best fit logistic regression model for word accuracy contained a main effect of Block (with the later block having lower accuracy), and an interaction between Word Age and Trial: Younger words improved in accuracy as trials progressed (β = 0.30802, Std. Error = 0.13558, z = 2.272). Critically, there was no significant interaction between Word Age and Pairing, although the numbers trended in the predicted direction (Table 1), carried by higher accuracy to young words when REAL and YOUNGER were paired. Participant Age was not significant, either in isolation or in an interaction.

**Table 1:** Mean accuracy rates and standard deviations (by speaker) for different word types across different real word pairings in experiment 1.

	YOUNGER-REAL	OLDER-REAL
Young Words	95.4% (5.8)	93.5% (7.9)
Old Words	93.5% (5.2)	93.6% (5.8)

#### Reaction times

Our reaction time analyses exclude incorrect trials. The dependent variable in our reaction time analyses is log RT. The best model of experiment 1 reaction times is shown in Table 2.

**Table 2:** Summary of best fit-model for response times in experiment 1.

	Estimate	Std. Error	t value
(Intercept)	6.577906	0.026993	243.69
Pairing = young-real	-0.04229	0.015501	-2.73
Word = young	-0.01137	0.017696	-0.64
Participant = older	0.08672	0.046691	1.86
Trial	-0.01304	0.003316	-3.93
Block = 2nd	0.041444	0.010498	3.95
Pairing = young-real x Word = young	-0.03501	0.016798	-2.08
Pairing = young-real x Participant = older	-0.02775	0.028135	-0.99
Word = young x Participant = older	-0.03572	0.028526	-1.25

**Fig 1:**
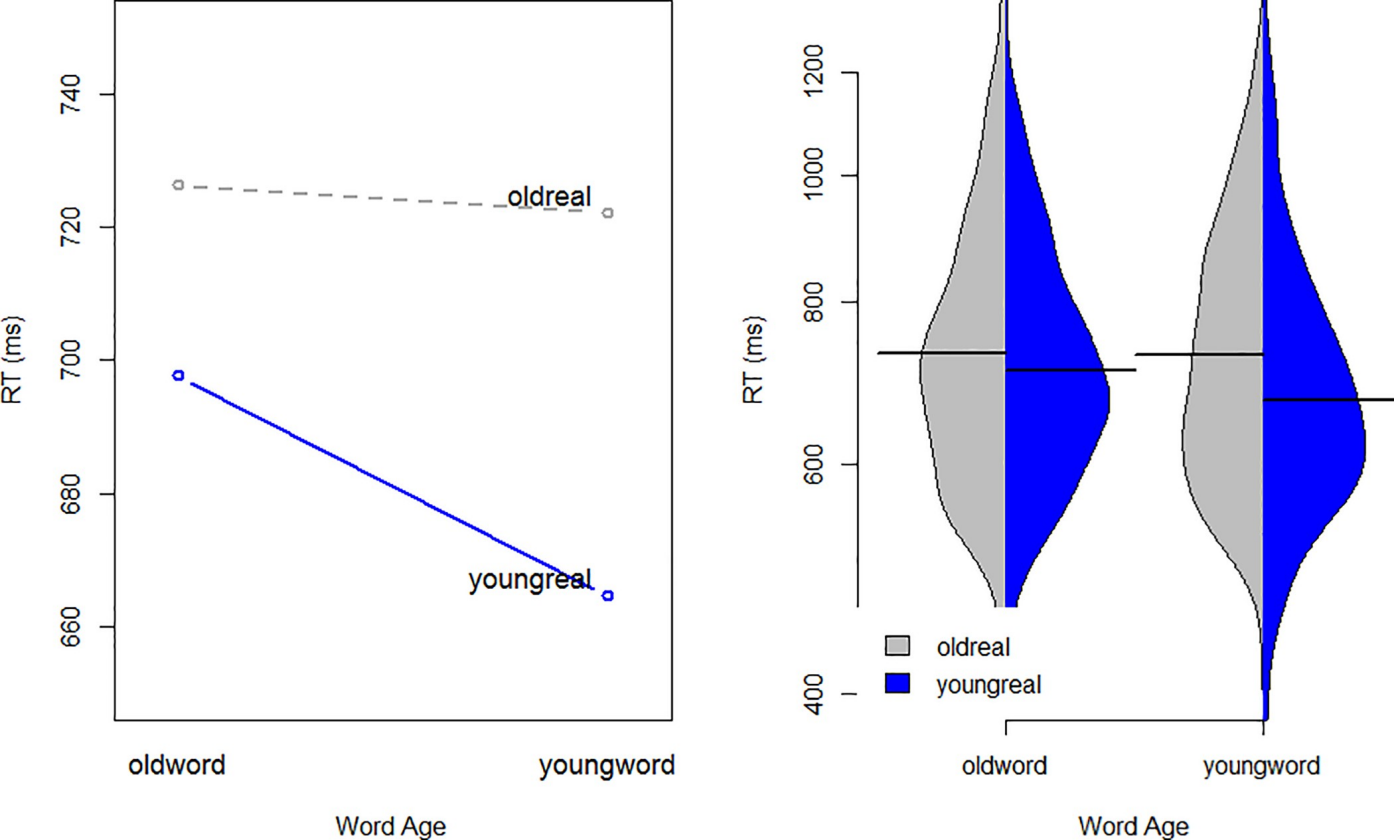
Interaction between word type and IAT pairing (condition) for younger participants only (born > 1991). Left panel shows model predictions, and right panel shows raw distributions of responses.

Like the accuracy model, we see a main effect of Block (participants are slower in the second critical Block), and Trial (participants get faster within a block as it progresses). There is also a significant three-way interaction between Pairing, Word Age, and Participant Age: younger participants show the predicted association between young words and young faces, but older participants do not. In order to investigate the nature of this interaction further, we fit two separate models, one to the older participants and one to the younger participants. The pairing by word-type interaction did not reach significance for the older participants, but it did reach significance for the younger participants (β = -0.042924, Std. Error = 0.018292, t = -2.35). Fig 1 shows this interaction for younger participants: they are generally faster when YOUNG and REAL are paired, but significantly more so for words that are used more often by younger speakers. However, responses to old-words do not become faster when paired with OLD faces. Note that our RT models all use log RT as the dependent variable, but in our figures we use an exponential function to convert model predictions back to RT, for interpretability.

### Summary of experiment one

This study complements the findings of Walker and Hay [3] by showing that listeners appear to be sensitive to the aged distribution of words, since there is evidence that they implicitly associate younger words with younger faces. The evidence for this effect comes from the reaction time data, and from younger participants only. There is also a more general effect that participants (older and younger) are faster when REAL and YOUNG are paired, replicating Rothermund and Wentura’s findings [51, 53], and supporting their argument that salience congruence may account for some of the facilitation in the IAT.

While younger participants were significantly faster responding to young-words compared to old words when REAL and YOUNG were paired, there was not the predicted crossover effect for old-words when REAL and OLD were paired: access of old-words was not facilitated by a “congruent”-face pairing. It’s possible that this is due to the general difficulty participants have in pairing OLD and REAL (compared to YOUNG and REAL) [51, 53], which is delaying lexical access more generally and cancelling any facilitation that might come from congruence. That is, the lack of effect isn’t because of a lack of an association between old faces and old words, but a product of this specific methodology, and the fact that there was a crossover effect using these same words in [3] supports this interpretation. The alternative is that this asymmetry in our results reflects a true asymmetry in lexical representation and/or access: our young listeners might attend differently to words coming from older and younger speakers, and encode stronger relationships between young-faces and young-words than they have been old-faces and old-words. Such an account would be consistent with recent work highlighting the ways in which raw experiences are encoded differently depending on social factors [32].

We did not hypothesize a distinction between the younger and older *participants* in terms of the Word Age and Pairing interaction, and so we would need to replicate these results before assigning much weight to them. There is certainly evidence in the social psychology literature that older and younger people differ in their conceptualizations of their own age, and age more generally [66, 67], and evidence from studies of language change suggests that young people, especially adolescents, are most heavily invested in aged-based language differentiation [68,69]. Therefore, it is conceivable that our older and younger participants either differed in how they responded to the labels and faces used within our task, or in how they socially encoded the words used in our study, and future work designed to test these hypotheses could be illuminating.

In experiment 2, we use the same methodology, but substitute the social category of age with gender. Gender has been identified as one of the most robustly attended to sociolinguistic categories; gendered variants in speech arise early, are attended to early [70], and adult learners are easily able to learn new linguistic associations with gender [71]. Thus, if there is any social generalization that is most robustly associated with word use, we should predict that it would be gender.

## Experiment 2: Female and male words, female and male faces

In this experiment, we test for an implicit association between words used more often by men/women and male/female faces. The design is similar to experiment 1, except we used words that are biased in production frequencies such that they are used more often by men than women, or vice versa (as opposed to older and younger speakers). The purpose of this experiment is to conceptually replicate experiment 1, using a different social category of speakers.

### Methodology

#### Stimuli

The stimuli in this experiment consisted of photographs of male and female faces, and single words in text. The 100 words in this study consisted of 50 real words and 50 non-words. The real words were overrepresented in the speech of either male or female speech in the Canterbury Corpus [57]. Of the 815,000 transcribed words in the corpus, 401,188 come from male interviewees, and 416,055 from female interviewees. In choosing real words that were skewed in their usage by men or women, we avoided names, function words, and expletives, with a preference for words that had higher overall frequencies. 25 words that skewed female in their usage were selected, which were between 32:1 (*pony*) and 1.9:1 (*class*, *teacher*) times more common in the female vs. male corpora, and had an in female-corpus frequency range of 31 to 827 ppm (parts per million). Words that had a frequency less than 45 ppm in the female corpus all had ratios of at least 7:1, so that the less skewed ratios were in more frequent words where we might expect participants to have more exposure to the (albeit weaker) bias. The 25 male words ranged from being around 20:1 (*vehicle*) to 1.97:1 (*guys*) times more common in the male versus female corpus, and had a frequency range of around 32–364 ppm in the male corpus. Again, words with a ppm in the male corpus less than 45ppm all had ratios of at least 7:1. There was no significant difference between the frequency of male words in the male corpus (*mean* = 115.26ppm, std.dev = 113.94), or female words in the female corpus (*mean* = 215.38ppm, std.dev = 238.22) in a Wilcoxon rank sum test (W = 282, p = 0.4227).

The 50 non-words were created from the real words, maintaining stress pattern, syllable structure, and orthographic length. Due to experimenter error, only 49 non-words were presented in Blocks 2 and 4. See Appendix B for the list of real and non-words. In a rating task that followed the main experiment, participants were asked to rate the real words on a 5-point scale of age usage, from 1, meaning that this words is “more frequently used by males” to 5, meaning this words is “more frequently used by females”. Male words received an average rating of 2.58 (sd = .49), and female words received an average rating of 3.53 (sd = .46). A Wilcoxon rank sum test found this difference in overt perceptions of word use depending on gender to be significant (W = 574.4, p < 0.001). These words thus differ from the age-graded words, in that participants have greater conscious awareness about the gendered distribution of these words.

While we already knew that the distributional age-skew of the words used in experiment 1 affected participants behavior in a lexical decision task, since the same words had been used in [3], no such prior experiment existed for gender-skewed words. Therefore, we conducted a lexical decision task analogous to [3], where participants were presented with the male words, the female words, 50 gender-neutral words (in terms of their distribution), and 100 nonsense words. The words were recorded by a male and a female, who were both native speakers of NZE and in their early 20s. Twenty-three native speakers of NZE took part in the study (7 M, 16 F). Stimuli for the experiment were divided between two blocks. Each block contained 200 words, split equally between the male and female speaker, and no words repeated within block. Therefore, each participant heard all words twice, once from each speaker across the two blocks (with order counterbalanced across participants). While there was no effect of voice-gender and word-gender congruence on error rates, an analysis of response times to correct answers showed a) that listeners were faster overall with the female compared to the male speaker, and b) that this advantage was largest in words that were said more by women in the CC. These results suggest that the gendered distribution of these words also affects lexical access, in a similar way to the age-distribution of words used in experiment 1.

To produce the photos, five females and males were recruited, and their photographs were taken and morphed into novel faces as outlined in experiment 1. Specifically, each original face was morphed with another face of his or her own sex to create a unique face. In total, 20 unique photographs were created depicting unique faces (10 female, 10 male). All photos appeared to be natural to the authors (examples available at https://github.com/jenniferhay/hayetal-plos2019/supplementaryfigures.pdf).

#### Participants

Fifty participants (32 female, 18 male) were recruited at the University of Canterbury, and compensated with a $10 voucher for their time. Participant age ranged from 18–56 years (median = 21). Forty-two of these participants also did experiment 1, prior to experiment 2, in the same experimental session. For experiment 2, we purposely aimed to increase the number of male participants, in order to increase comparability across male and female participants. It thus contains 8 additional males. All participants were native or near-native speakers of New Zealand English (defined as having moved to New Zealand before the age of seven).

#### Procedure

The experimental procedure was the same as experiment 1, except for where participants sorted faces into older and younger categories in experiment 1, they now sorted faces into male and female categories.

### Results

Model fitting proceeded as described for experiment 1, but substituting participant gender for participant age, and word gender for word age. The same outlier removal process was used as for experiment 1, and removed 5.5% of the data.

#### Accuracy

The overall accuracy rate was 94.6%. Table 3 shows the mean accuracy rates by hand-pairing and word type, and shows that accuracy is higher when the gender of the photo is aligned with word gender: subjects are more accurate when responding to male words when using the same hand to respond to male photos than when using that hand to respond to female photos. When responding to female words, they are most accurate when using the same hand as they are using to classify female photos. The best model of response accuracy to words is shown in Table 4. The model shows an effect of trial (with accuracy decreasing as trials progress), and the predicted interaction between word gender and photo-word pairing.

**Table 3:** Mean accuracy rates and standard deviations (by speaker) for different word types across different real word pairings in experiment 2.

	FEMALE-REAL	MALE-REAL
Female Words	95.0% (7.4)	93.2% (7.0)
Male Words	91.6% (8.9)	94.2% (6.9)

**Table 4:** Summary of best-fit model for accuracy rates in experiment 2.

	Estimate	Std. Error	z value
(Intercept)	3.5711	0.2923	12.219
Participant = male	0.6446	0.2589	2.49
Word = male	-0.7908	0.303	-2.61
Pairing = male-real	-0.7363	0.3063	-2.404
Trial	-0.1308	0.0462	-2.832
Word = male x Pairing = male-real	1.0893	0.4081	2.669

#### Reaction times

**Fig 2:**
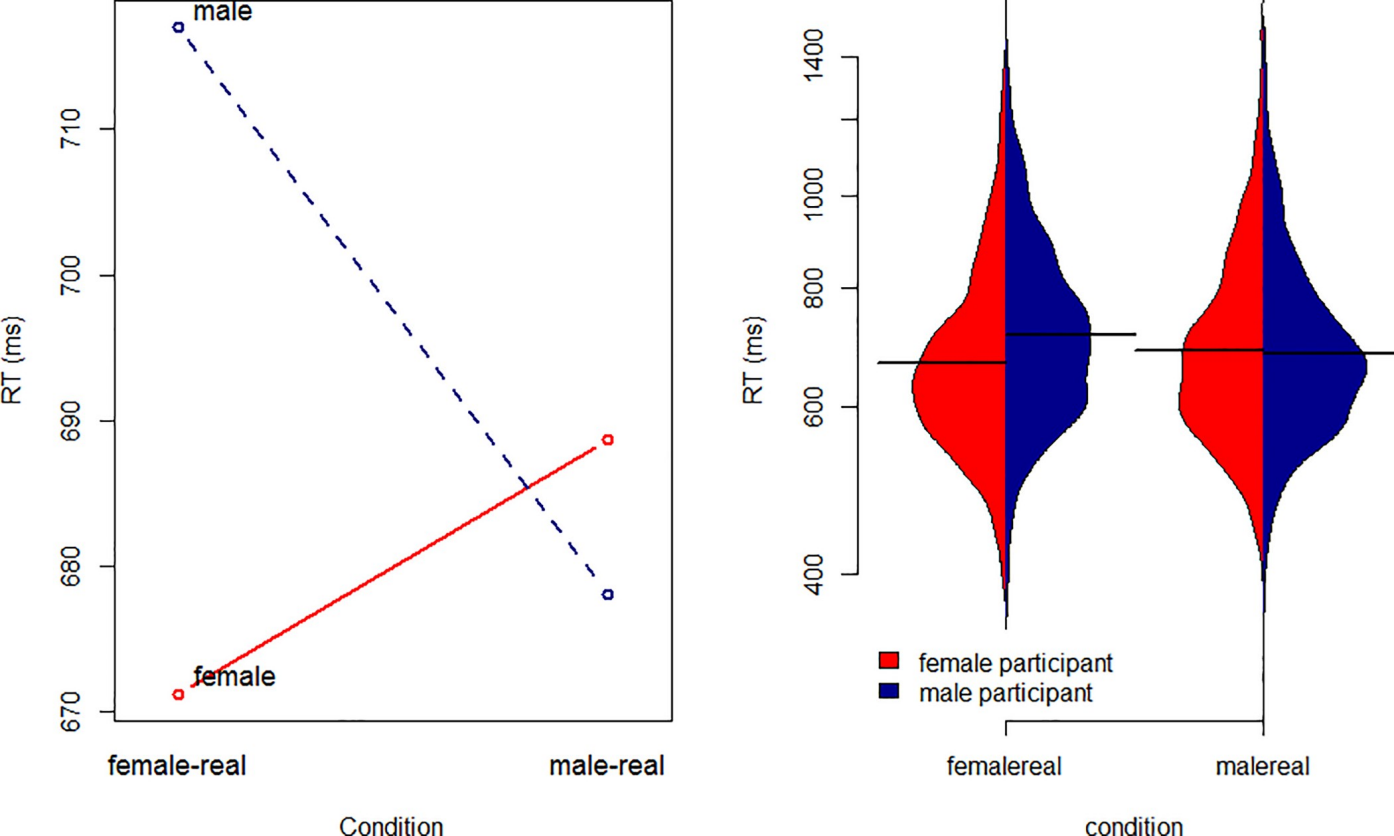
Model prediction for response times in experiment 2, as a function of word-pairing and participant gender.

The best model of the logged RTs to correct words is shown in Table 5. It includes 2 two-way interactions. The first shows that male participants do not speed up as much during the course of the experiment as female participants. The second shows that male participants are faster in the MALE-REAL pairing, and female participants are faster in the FEMALE-REAL pairing, as shown in Fig 2. Like the facilitative effect of pairing YOUNG and REAL or OLD and NOT REAL in experiment 1, this would appear to reflect a general benefit of pairing an unmarked social category (here, the participant’s own gender) with real words, and a marked social category with nonsense words [54]. There is critically no effect of word-gender on response times.

**Table 5:** Summary of best-fit model for response times in experiment 2.

	Estimate	Std. Error	t-value
(Intercept)	6.510292	0.022264	292.41
Participant = male	0.064969	0.036672	1.77
Pairing = male-real	0.025687	0.010868	2.36
Trial	-0.022376	0.002722	-8.22
Participant = male x Pairing = male-real	-0.081482	0.017757	-4.59
Participant = male x Trial	0.018451	0.004479	4.12

### Experiment 2 summary

Like experiment 1, experiment 2 suggests that participants have an association between words that are more likely to be used by a group of speakers–here women and men–and those groups, suggesting that listeners are tracking distributions of usage. In experiment 2, the predicted result is apparent in the accuracy data, but not the RT data, whereas in experiment 1, the accuracy data trended in the same direction, but it was the RT data that reached significance. Since both accuracy and response times reflect lexical access, we think the two experiments support an interpretation consistent with the hypotheses under investigation: listeners associate words that are used more often by certain groups of speakers with photos of faces representing those groups of speakers.

Since the photos and the words do not co-occur in presentation (the image of the face is not on the screen at the same time as the word is presented), this already suggests a more abstract relationship between speaker gender and word gender than demonstrated in our earlier work [3]. However, since it is quite likely that because people hear these words more often coming from female and male speakers, their memories of the words not only include acoustic traces of female and male voices, but also include visual information, including faces of the speakers. This means that we might also get the observed effects in a purely exemplar account of language, since female words would have been encountered more with female faces. In experiment 3, we therefore explore whether we can replicate the effect that we have seen in experiment 2, but instead of using faces, we ask participants to classify stereotypically gendered *objects* as either male or female.

## Experiment 3: Female and male words, female and male objects

Experiment 2 (and the associated lexical decision task), indicate that congruence between a person’s perceived gender (through voice or face) and the gendered distribution of word use facilitates lexical access. Both voices and faces are involved in the speech experience, however, so a congruency effect might arise in the absence of any abstracted generalization regarding gender. In experiment 3, we examine the relationship between words and the gender (female, male) of people who use those words most often by testing for an implicit association between words and a more secondary gender association via objects. That is, we get more abstract by using objects that have themselves been associated more with females or males, but aren’t necessarily directly associated with memories for the particular words we are interested in. If this effect is observed, then this would provide strong evidence that our observed associative effects are being driven by higher-order contextual labelling of individual speech events, where words and items are being categorized in (and abstracted to) the larger category of either FEMALE or MALE. We set out to test this in experiment 3, by replacing the faces used in experiment 2 with photos of gendered objects.

### Methodology

#### Stimuli

The stimuli for experiment 3 consisted of the same 50 gendered words, and 50 non-words, from experiment 2, and a set of 20 photographs of objects (10 female, 10 male). Each photo depicted a single object in the center of the image on a plain white background. Photographs were obtained via the creative commons search function (http://search.creativecommons.org/). Photographs of items were selected to be highly gendered and matched according to similar categories (e.g. category “bag” for women was represented by a purse, while for men it was represented by a briefcase). The colours in the two sets are also highly gendered, with female objects predominantly presented in red and pink, and male objects predominantly in darker colours such as blue and black. The genderedness of the photos then, is carried both by the object type and the color of the object.

**Fig 3:**
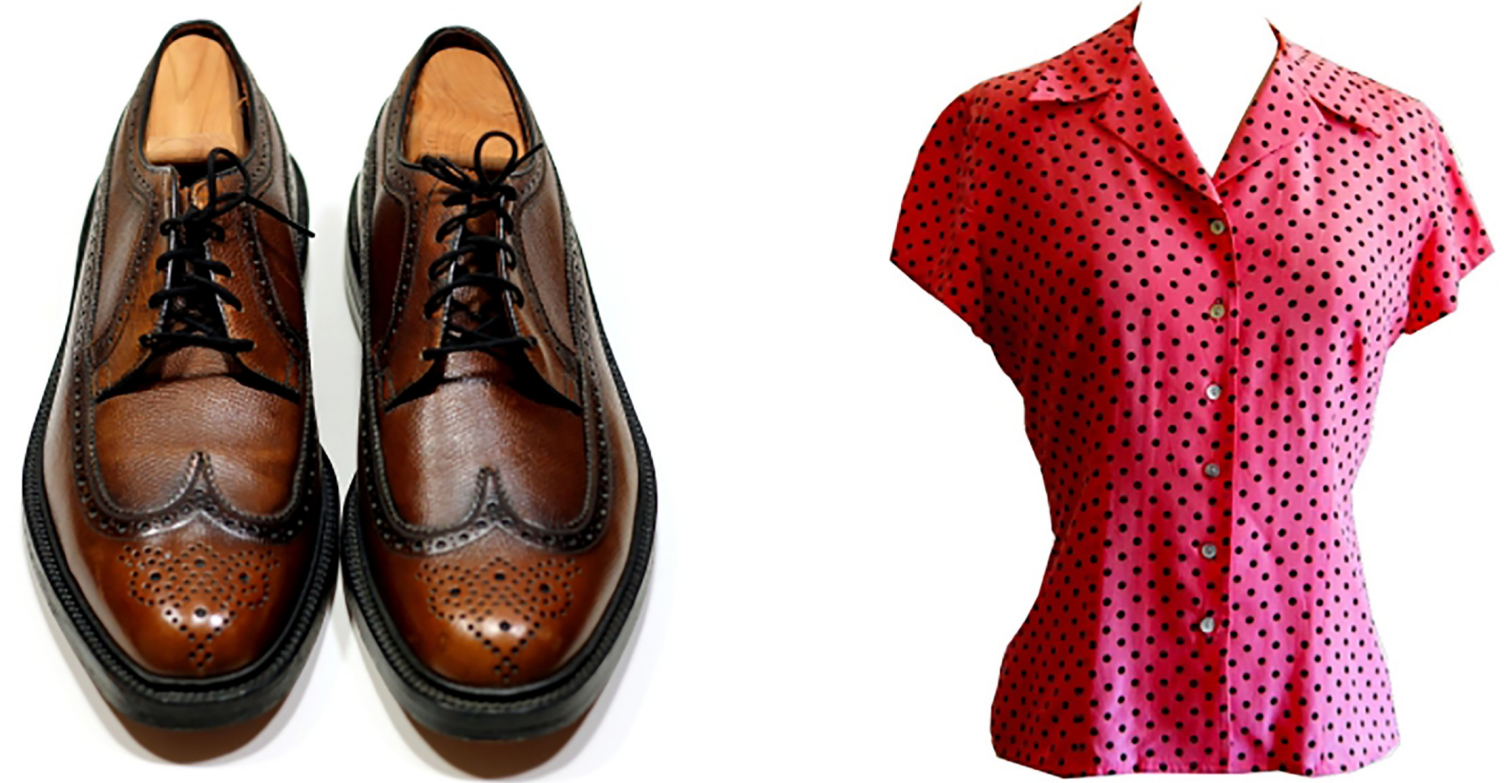
Two images used in experiment 3. (Photos: Sheie, R. (2011). Wingtip [Online image]. Creative Commons Attribution License (CCAL) CC BY 4.0. Retrieved from https://www.flickr.com/photos/85546319@N04/8444009146/in/set-72157631599543637 Vintage 1980s pink polka dot silk blouse [Online image]. (2010). Licensed under Creative Commons Attribution License (CCAL) CC BY 4.0. Retrieved from https://www.flickr.com/photos/huzzahvintage/4664390710/).

To ensure that the photographs were appropriate for the experiment, a larger subset of candidate photos were rated by 12 participants, aged 20–35. These participants were asked to address how gendered the items were, how recognizable the items were, and how semantically matching the pairs of items (i.e., purse, briefcase) were. The items per category and gender with the highest approval rating were then selected for the experiment. See Fig 3 for examples of the pictures used in experiment 3 (the full set of pictures is available at https://github.com/jenniferhay/hayetal-plos2019/supplementaryfigures.pdf.

In an attempt to minimize non-hypothesized effects of increased performance when the word *real* is paired with an ingroup, or an unmarked category, in experiments 3 and 4 we used the labels ‘Word’ and ‘Non-Word’ as opposed to ‘Real’ and ‘Not Real’.

#### Participants

Twenty-eight (22 female, 6 male) participants were recruited at the University of Canterbury, and compensated with a $10 voucher for their time. Participant age ranged from 18–40 years (median = 20). All participants were native or near-native speakers of New Zealand English (defined as having moved to New Zealand before the age of seven).

#### Procedure

The experiment was almost identical to experiment 2, except that where participants had been presented with male and female faces to sort into MALE and FEMALE, they were now presented with the photos of stereotypically male and female objects. They responded to the same words as in experiment 2, and categorized them as WORD or NON-WORD. The experiment was run by the third author (a young, female, native speaker of American English).

### Results

The reaction times and errors rates were analyzed using the same types of statistical modeling as in experiment 2, with the same factors tested (except faces were replaced with objects this time). The outlier removal procedure resulted in removal of 5.8% of the data.

#### Accuracy

The overall accuracy rate was 91.6%, and mean accuracy rates by word type and pairing are presented in Table 6. As with the gendered faces, there is an interaction between word gender and photo-word pairing, such that participants are more accurate at responding to words when the word gender matches the photo-word pairing. This interaction is significant in the best model of accuracy is shown in Table 7. The only other significant effect is an effect of trial, with participants’ accuracy decreasing as each block progresses.

**Table 6:** Mean accuracy rates and standard deviations (by speaker) for different word types across different real word pairings in experiment 3.

	FEMALE-REAL	MALE-REAL
Female Words	95.4% (5.7)	87.5% (11.4)
Male Words	91% (6.7)	92.6% (8.5)

**Table 7:** Summary of best-fit model for accuracy in experiment 3.

	Estimate	Std.Error	z-value
Intercept	4.36179	0.49820	8.755
Word = Male	-1.80018	0.48259	-3.73
Pairing = male-word	-1.64027	0.48684	-3.369
Trial	-0.13006	0.04839	-2.688
Word = male x Pairing = male-word	2.51091	0.54149	4.637

In order to test whether there are any significant differences between the effect size in experiments 2 and 3 –i.e., whether faces or objects resulted in stronger effects [37]–we combined the data from both experiments. We started by testing for all 4 way interactions between Experiment, Word Gender, Pairing and Participant sex, and then pruned the model down until all factors were significant. In the combined data-set, Word Gender x Pairing remains highly robust (Word = male x Pairing = male-word: β = 1.615, Std. Error = 0.31674, z = 5.099), but Experiment does not reach significance in isolation, in pairwise interactions with Word Gender or Pairing, or in the critical three-way interaction. This suggests that the effect of Word Gender x Pairing on accuracy is similar whether the gendered images being sorted are faces or objects. Interestingly, the combined model also shows an accuracy advantage when the participant sex and the pairing are matched, as was observed in RTs in experiment 2 (Participant = male x Pairing = male-real/word: β = 1..09, Std. Error = 0.32552, z = 3.376). This does not interact with Experiment, and we thus assume that it emerges in this combined data-set due to the increased sample-size.

#### Reaction times

**Fig 4:**
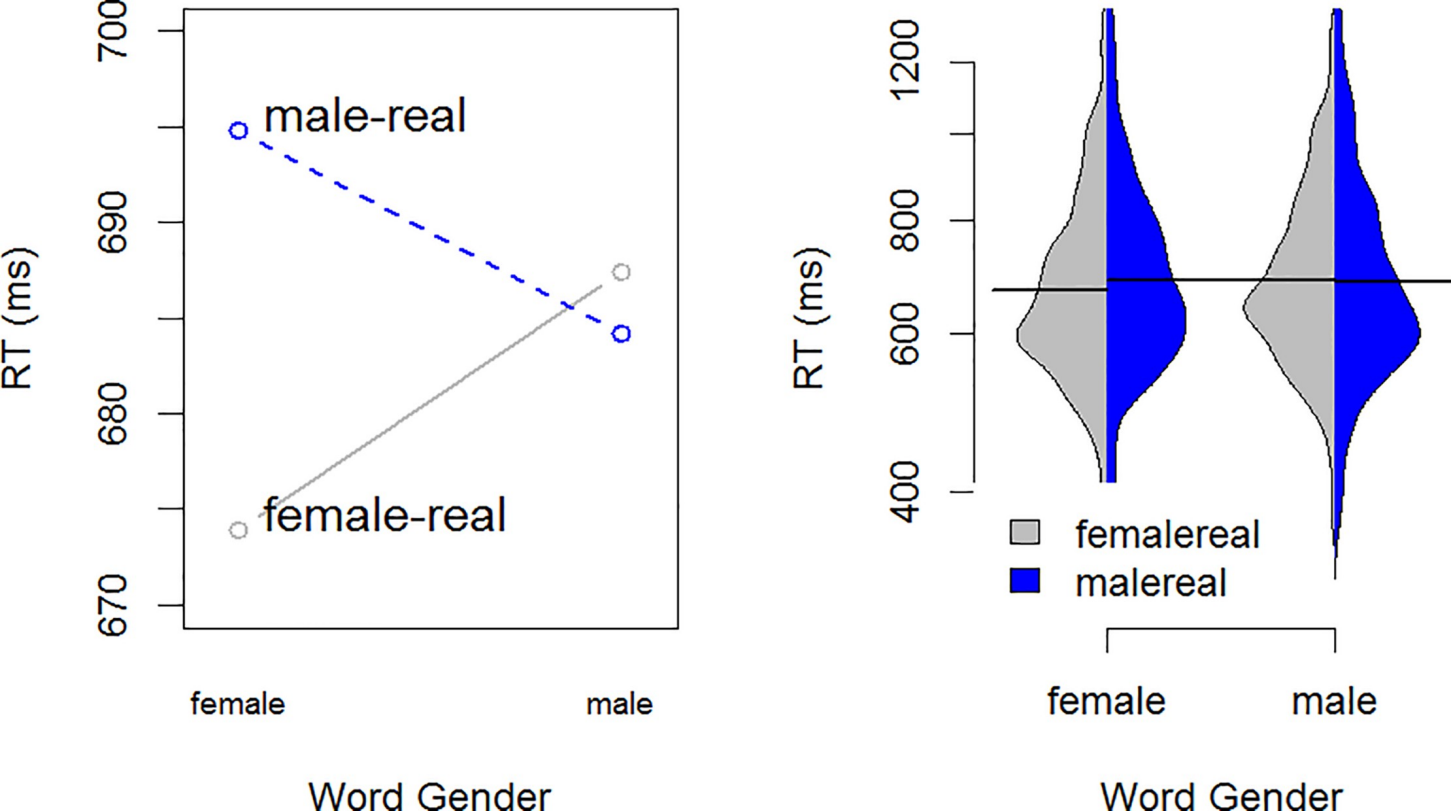
Model predictions of response times by word gender and IAT pair in experiment 3.

The dependent variable in our reaction time model was log transformed reaction times. The best model is shown in Table 8. There was an unanticipated interaction between trial number and word gender, with speed increasing through the course of each block more dramatically for female words (a decrease in RT of ~60ms) than male words (~20ms). The hypothesized interaction between photo-word pairing and word gender was significant, and is plotted in Fig 4: female words are responded to more quickly when WORD is paired with FEMALE and male words are responded to more quickly when WORD is paired with MALE.

**Table 8:** Summary of best-fit model for reaction times (logged) in experiment 3.

	Estimate	Std. Error	t
(Intercept)	6.513535	0.027107	240.29
Word = male	0.019454	0.014083	1.38
Pairing = male-word	0.030516	0.015595	1.96
Trial	-0.02672	0.003729	-7.17
Word = male x Pairing = male-word	-0.03519	0.016075	-2.19
Word = male x Trial	0.020871	0.00528	3.95

Recall that the predicted reaction time effect was not present in experiment 2. In order to establish whether there is a genuine difference between the objects and faces in terms of our predicted effect, we fit a combined model of experiments 2 and 3. We started by testing for all 4 way interactions between Experiment, Word Gender, Pairing and Participant sex, and pruned the model down until all factors were significant. The resulting model did not include Experiment x Word Gender x Pairing, suggesting there is no robust difference between the experiments in our predicted effect. It does show a main effect of Word Gender x Pairing (Word = Male x Pairing = male = real/word, β = -0.020445, Std. Error = 0.010541, t = -1.94), interacting with trial, with the predicted effect present at the beginning of each block, and not at the end (Word = Male x Pairing = male = real/word x Trial, β = 0.016013, Std. Error = 0.006725, t = 2.38). The combined model retains an overall effect of participant sex, in which males are faster in the male-real/word pairing in both experiments (Participant = male x Pairing = male-real/word, β = -0.061041, Std. Error = 0.015764, t = -3.87).

### Experiment 3 summary

When using gendered objects instead of gendered faces, we have found evidence that listeners still associate words used more by women or men with FEMALE and MALE categories respectively. Since it is unlikely that participants encountered these words with the objects, this effect is unlikely to be attributed to visual information that is stored together with the words. Rather, it appears that the gendered objects, and the gendered words, share an association with MALE or FEMALE as categories that are abstracted away from specific memories of people.

Unlike experiment 2, we do not find evidence that participants do better when their own gender is paired with real words. However this is likely due to the fact that there were fewer men in experiment 3, as confirmed by the fact that there is no significant difference between the experiments in a combined model.

## Experiment 4: Female and male words, female and male objects, covert gender match

Experiments 2 and 3 demonstrate that people have associations between MALENESS and FEMALENESS and words that are used more by men and women. In both studies, the IAT task involved sorting gendered images (faces or objects) into MALE and FEMALE categories, making gender an explicit, conscious part of the task.

In our final experiment, we test whether the observed facilitation for object-word congruence depends on using the categorical labels MALE and FEMALE in the task by removing these labels from the task. By giving participants the labels MALE and FEMALE, we explicitly introduced gender into the task, and our results could reflect priming from the labels (rather than the faces/objects), and/or task-specific strategies by participants (i.e., “This task is easier if I think of these words as gendered”). Just as seeing toy Koalas and Kangaroos affected participants vowel perception in [41] despite no mention of AUSTRALIA, we wanted to see if our participants associated feminine objects with female-words, without mentioning FEMALE.

We do this by removing the MALE and FEMALE labels from the sorting task, and instead using a more covert tactic: images of gendered objects are presented with or without frames, and participants sort images depending on whether an image is FRAMED or UNFRAMED. For half the participants, FRAMED is exclusively associated with female objects, and UNFRAMED with male objects, and if participants associate these objects with female/male words respectively (through their internal social labels of MALE and FEMALE), we would expect them to be faster at accessing female words when sorting FRAMED and REAL with the same hand. We also predict that this effect might take a while to emerge, as participants consciously or subconsciously activate the gendered associations of the objects.

### Methodology

#### Stimuli

**Fig 5:**
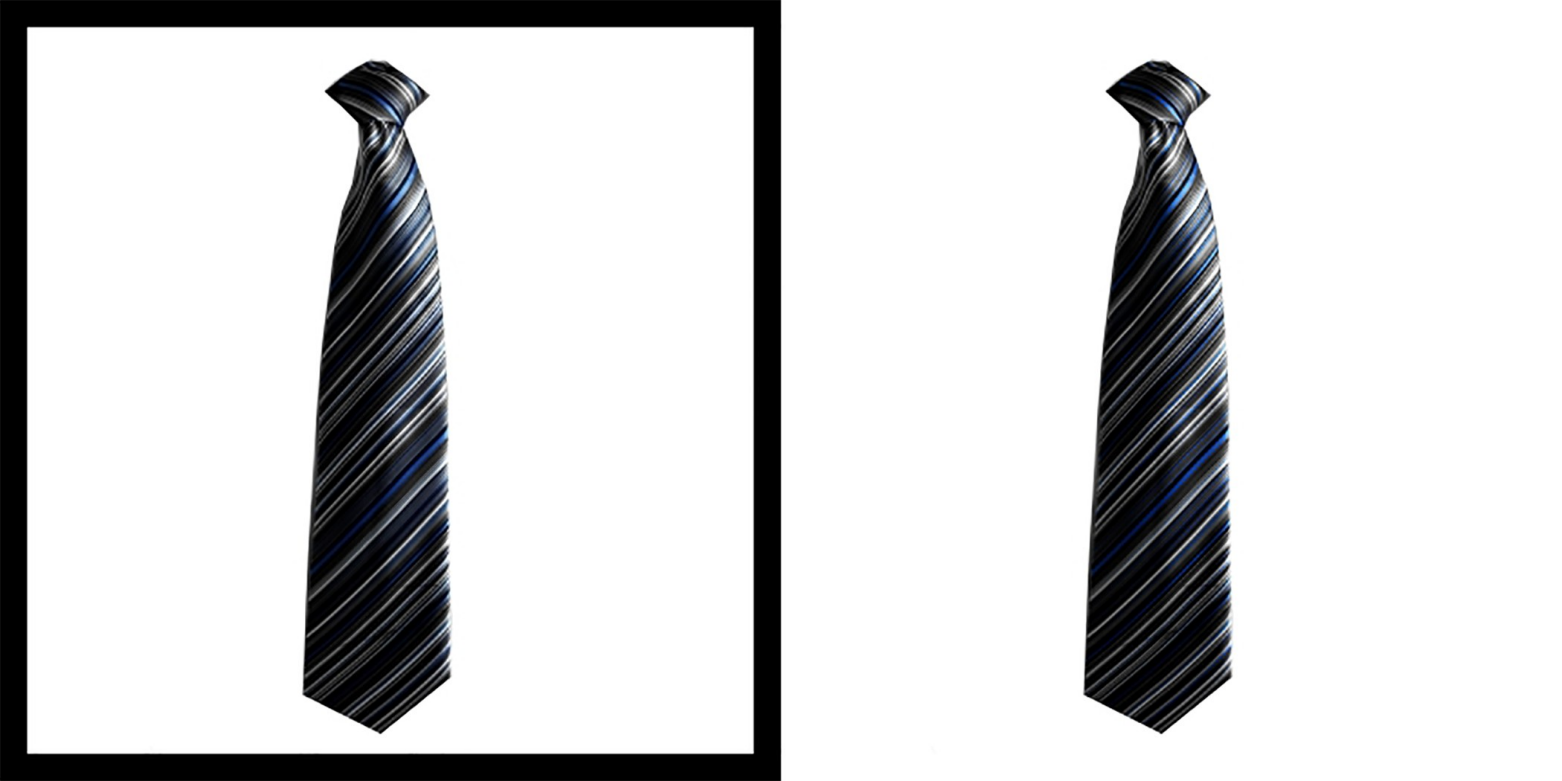
**Unframed (left) and Framed (right) version of an image used in experiment 4**. (Photo: Bennett, A. (2011). Blue [Online image]. Licensed under Creative Commons Attribution License (CCAL) CC BY 4.0. Retrieved from https://www.flickr.com/photos/beeny87/5878115657/).

The words used in experiment 4 were the same gendered and non-words used in experiments 2 and 3. The images used were the same object images used in experiment 3. In this experiment however, the images either appeared with or without frames around them (see Fig 5).

#### Participants

The participants in this study are the same participants used in experiment 3. They completed experiment 4 before experiment 3 (so that gender would not have been mentioned yet), in the same experimental session.

#### Procedure

The procedure was the same as the preceding experiments, except that instead of sorting images into male and female categories, participants sorted images based on whether they had a frame around them or not (see Fig 5). Framed items were always linked with one gender and paired with the WORD label. Half of the participants experienced the female objects aligned with FRAME and WORD in the first part of the experiment and the male objects aligned with FRAME and WORD in the later blocks. This order was reversed for other participants.

### Results

The same model fitting procedure was used as in the previous experiments. The outlier removal process resulted in removal of 4.8% of the data.

#### Accuracy

**Fig 6:**
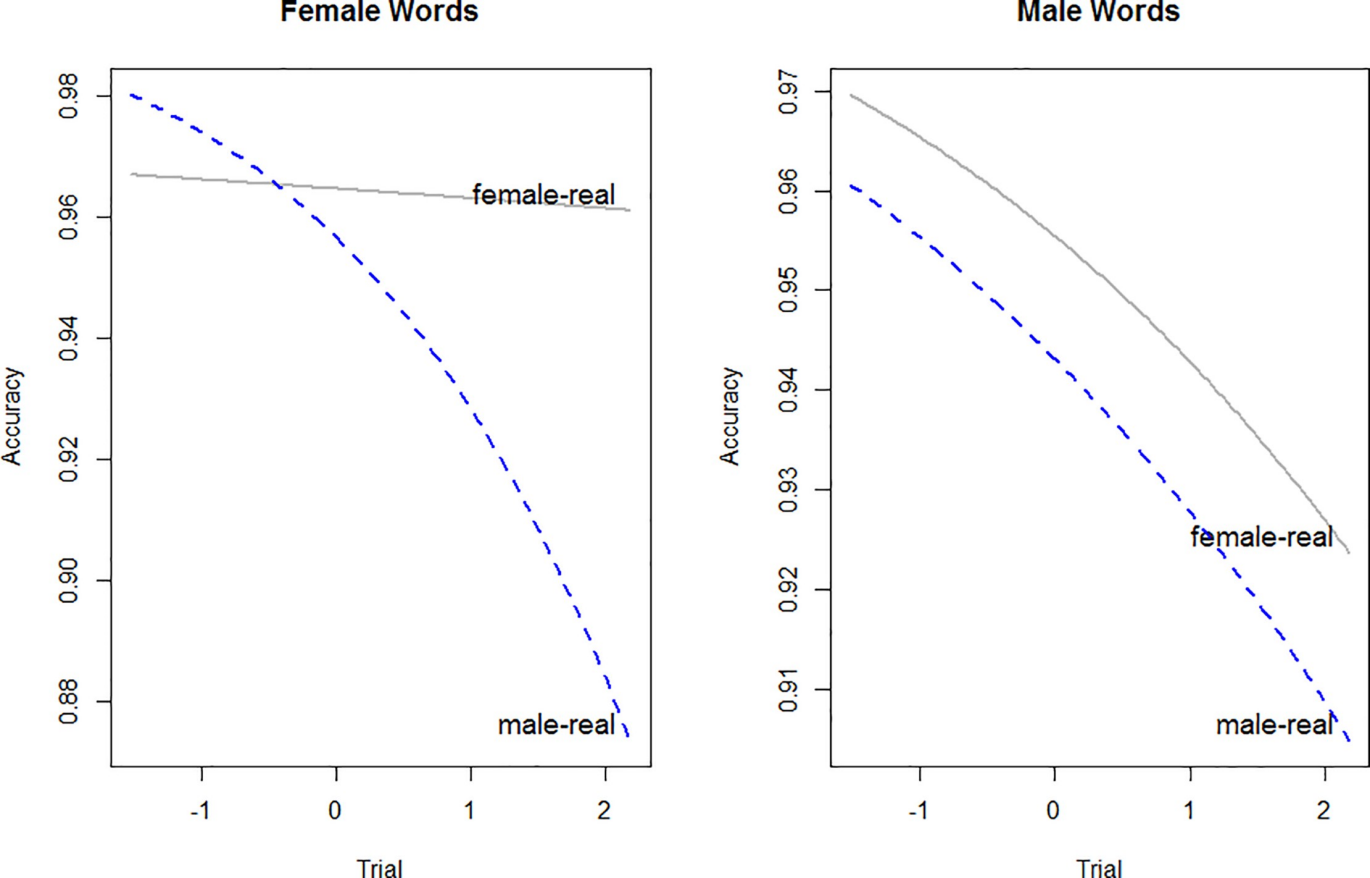
Experiment 4. Three-way interaction between trial, pairing and word gender. The female words are shown in the left panel, and male words are shown in the right. The female-real pairing is shown in gray and the male-real pairing is shown in blue.

**Fig 7:**
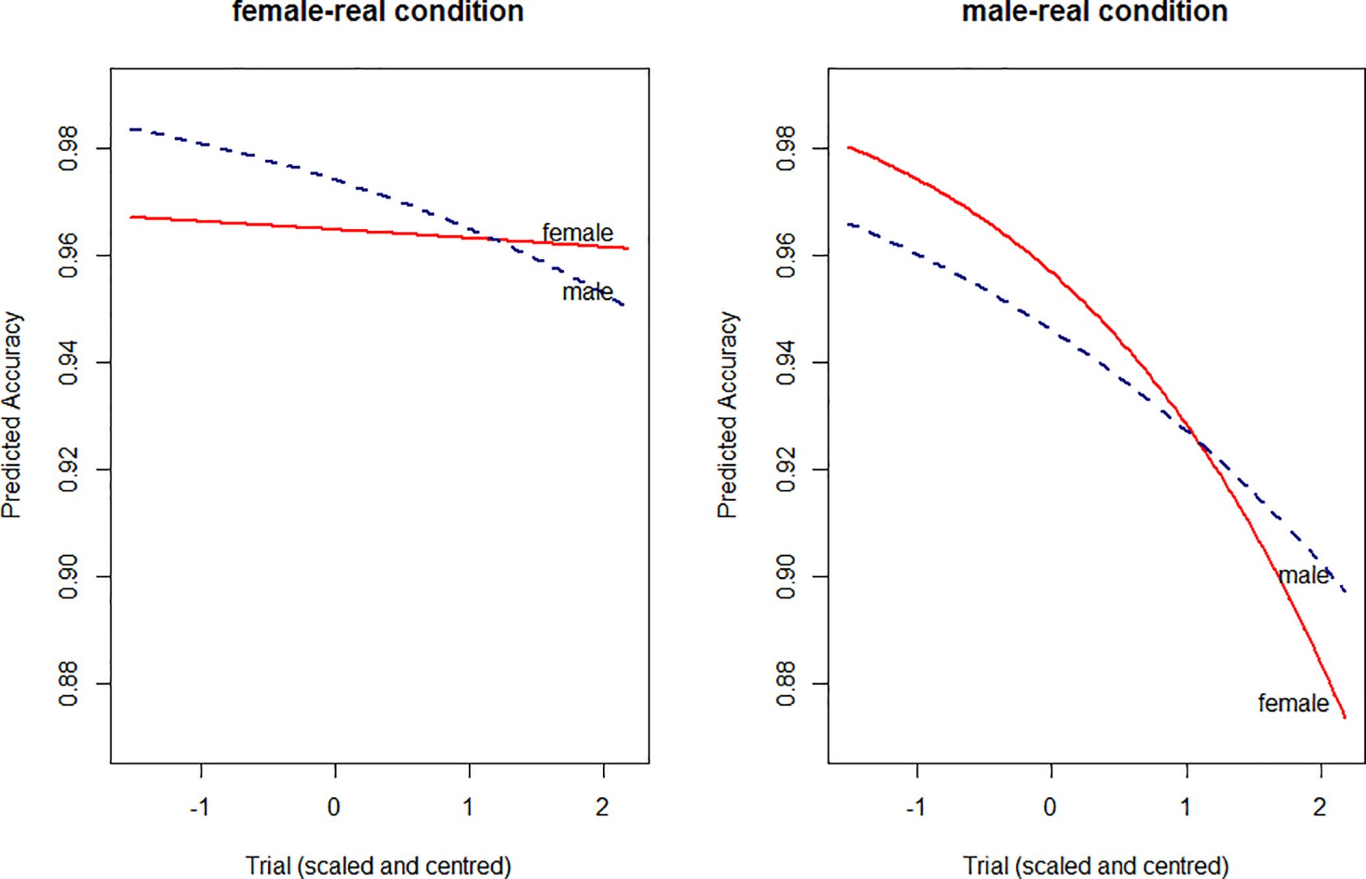
Three-way interaction between trial, pairing and participant gender. The female-word pairing is shown in the left panel, and the male-word pairing is shown in the right. Female participants are shown in red, male participants are shown in blue.

The overall accuracy rate was 92.5%, and mean accuracy rates across word type and pairings are presented in Table 9. Participants are more accurate with female words in the FEMALE-REAL pairing, but not more accurate for male words in the MALE-REAL pairing.The best fit model for accuracy showed two three-way interactions, and is given in Table 10. The first was the interaction between Trial, Word Gender, and Pairing, as shown in Fig 6. This shows that accuracy decreased as trials progressed, except for female words in the female-word pairing, which remained highly accurate throughout. The second was an interaction between Trial, Participant Gender and Pairing, as shown in Fig 7. This interaction shows that male participants decreased in accuracy more dramatically than females in the FEMALE-WORD pairing, whereas female participants decreased more dramatically in the MALE-WORD pairing. It should be noted that the number of male participants in this experiment is not large (6 M, 22 F), and so this interaction, while significant, should be treated with some caution. If the model is fit without interactions involving Participant Gender, the more critical Trial, Word Gender, and Pairing remains intact.

**Table 9:** Mean accuracy rates and standard deviations (by speaker) for different word types across different real word pairings in experiment 4.

	FEMALE-REAL	MALE-REAL
Female Words	94.4% (5.4)	91.7% (7.3)
Male Words	92.5% (7.8)	90.7% (7.6)

**Table 10:** Model coefficients for best fit model of accuracy in experiment 4.

	Estimate	Std. Error	t
(Intercept)	3.30886	0.29893	11.069
Trial	-0.04585	0.11282	-0.406
Word = male	-0.24343	0.33755	-0.721
Pairing = male-word	-0.21244	0.30786	-0.69
Participant = male	0.31529	0.49763	0.634
Trial x Word = male	-0.2165	0.14536	-1.489
Trial x Pairing = male-word	-0.48826	0.15087	-3.236
Word = male x Pairing = male-word	-0.04529	0.33174	-0.137
Pairing = male-word x Participant = male	-0.54931	0.37092	-1.481
Trial x Participant = male	-0.26438	0.18842	-1.403
Trial x Word = male x Pairing = male-word	0.49592	0.19402	2.556
Trial x Pairing = male-word x Participant = male	0.4795	0.23858	2.01

#### Response times

**Fig 8:**
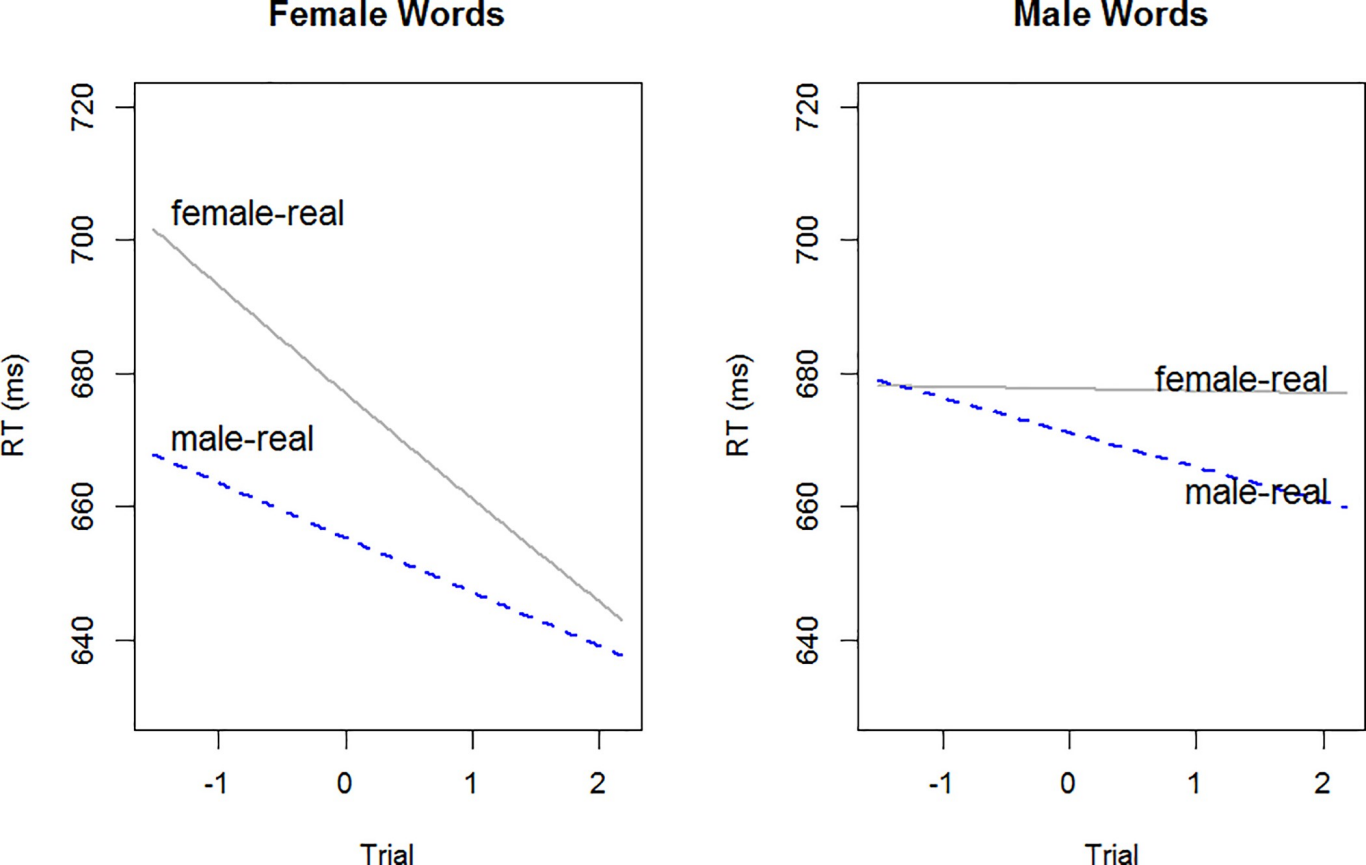
Experiment 4. Three-way interaction between trial, pairing and word gender. The female words are shown in the left panel, and male words are shown in the right. The female-real pairing is shown in gray and the male-real pairing is shown in blue.

The best fit model of log RT is presented in Table 11. It contains a 3 way interaction between Word Gender, Pairing and Trial. This is plotted in Fig 8. Female words decrease in RT throughout each condition, but do so fastest in the female-real pairing. Male words decrease in RT in the male-real pairing, but not in the female-real pairing. These results echo the accuracy results, in that there appears to be a learning effect, and that this is most apparent in the female words when in the female-real pairing. However they should also be interpreted with some caution, in light of the fact that there is an extra puzzling detail. While the female words increase in speed throughout the female-real pairing, they nonetheless remain slower in the female-real condition than in the male-real condition throughout. Inspection of the accuracy and the RT results together indicates that the female-real condition is, for some reason, overall more accurate, and with slower response times. The relevant aspect of the RTs result for our hypothesis is that RT speeds increase in this condition for the female words, but not the male words.

**Table 11:** Model coefficients of best fit model for response times in experiment 4.

	Estimate	Std. Error	t value
(Intercept)	6.5176	0.0231	281.83
Block = 2nd	-0.0220	0.0065	-3.36
Trial	-0.0237	0.0043	-5.55
Word = male	0.0010	0.0119	0.08
Pairing = male-real	-0.0325	0.0083	-3.94
Trial x Word = male	0.0232	0.0061	3.83
Trial x Pairing = male-real	0.0111	0.0061	1.82
Word = male x Pairing = male-real	0.0229	0.0100	2.29
Trial x Word = male x Pairing = male-real	-0.0184	0.0087	-2.11

### Experiment 4 summary

This experiment used the same stimuli and general procedure as experiment 3, where participants sorted gendered objects and words. However, unique to this experiment, participants did not explicitly sort the objects based on their gender, via labels of FEMALE or MALE, but instead sorted the objects based on whether they were framed or not, where all items related to a specific gender were framed or not framed. Despite the covert nature of gender sorting, we again find an interaction between word gender and object gender though the effect takes time to develop over the course of the experiment. We interpret this result as indicating that participants take time to learn (consciously or subconsciously) that the framed/unframed images are meaningfully gendered. This interaction is apparent both in accuracy and in RT.

## Discussion

This project aimed to investigate the implicit associations people have between word usage and social categories. Across four experiments, we found that participants associated words that they have experienced more from certain groups of speakers with abstracted social categories. In experiment 1, reaction times suggested that younger listeners had an association between words encountered by older/younger speakers and older/younger faces respectively. In experiment 2, accuracy rates suggested that listeners had an association between words encountered by female/male speakers and female/male faces, respectively. In experiment 3, we showed that listeners had an association between words encountered by male/female speakers and male/female *objects* respectively (in both response times and accuracy rates), and in experiment 4, we showed that we did not have to ask participants to sort things explicitly by gender to see this effect emerge in accuracy and in response times.

### Implications for theories of lexical representation and access

At the most basic level, these data support other accounts arguing that listeners are tracking information about speaker identity, consistent with exemplar models of speech processing [30]: if lexical access is facilitated when a word’s presentation is more similar to previous presentations of the word, that entails that the word’s cognitive representation is shaped by experience (i.e., the specificity effect). In the current study, words that our participants have heard–across their lifespans–more often from men or women were generally accessed faster and/or more accurately when paired with male or female images, respectively.

In Walker & Hay [3] we presented words in old/young voices to participants in an auditory lexical decision task, finding facilitation when the voice age was congruent with the age of those who most commonly use the word. We argued that these results suggest that acoustic traces of older/younger voices are stored in memory, and that hearing words in an older/younger voice facilitates lexical access because of an acoustic match (though we did not rule out the role of an indexical match): a bottom-up effect. In experiment 1 and 2, where we pair words with faces, an argument could be made that the facilitation observed is similarly due to a specificity effect, this time visual: participants have encountered these words most often being spoken by those with female/male faces. However, these two experiments were already more abstract than Walker & Hay’s work in a number of ways. First, the words were presented orthographically rather than auditorily. Since the categorization of words as old/young/male/female was based on spoken frequencies, it’s improbable that participants have *seen* these words more with male or female faces (and we know that modality is important for specificity effects [72]). Second, the faces and words were not presented simultaneously: at any moment, a participant was sorting a face *or* a word. Like other IAT work, then, the facilitation is assumed to be based on the shared categorical associations the faces and words have with the labels OLD or YOUNG or MALE or FEMALE. That is, beyond an acoustic or visual match, word access is being facilitated by an indexical match of a category of social type, for example, MALE.

This line of argumentation is more aggressively supported by experiment 3 where we find facilitation for visually presented gendered words when they are paired with images of stereotypical gendered objects. Unlike the images of faces, which commonly accompany our experience of auditory speech, the images of objects and images of words are even less likely to commonly co-occur, and the objects are unlikely to be stored in episodic traces stemming from the same event. That is, gendered objects are unlikely to be stored in a word’s representation. It is more likely that the objects and words overlap at a shared categorical level, accounting for facilitated lexical access of certain words (i.e. through the shared and activated categorical label like FEMALE).

In experiment 4, we found that we could also see an association between gendered words and gendered objects without explicit gendered category labels. Here, participants were asked to sort gender-stereotyped object images under a non-gendered label (i.e. FRAMED/UNFRAMED) where the framed objects were stereotyped objects associated with one gender and the unframed objects were always stereotyped objects associated with another gender. As participants progressed through an experimental block, the gendered category (i.e., FEMALE) became activated and in doing so facilitated lexical access of female words.

Together, we take these findings as further evidence for a hybrid model of lexical representation [24, 73]. Just as experience with particular phonetic realizations has been shown to generalize to other words containing the same phoneme [12], the consistent experience of certain words coming from certain speaker groups builds relationships between those words and more abstract social categories. Because of this, language processing is facilitated not simply by a direct match between the acoustic and visual context a word has been encountered in, but also by an indexical match between, for example, gendered objects and gendered words [41, 42].

While we have been using the terms “label” and “abstract social category” to describe this indexical link between a word and an object, we are not committed to whether this link is a cognitively real, phoneme-like label (i.e., MALE), or consists purely of “aggregates of traces acting in concert at the time of retrieval” which “represent the category as a whole” [7] (p. 411). Our words and objects are linked by the men and women who predominantly use them, but men and women do not necessarily have to be in the probe itself (an orthographically presented word without a speaker; a picture of a briefcase without a person holding it) to be activated through the similarity of the probe to memory traces which DO contain a person, without the need for a label like MALE [7]. One could argue that the fact that we see evidence for an association developing in experiment 4, in which we removed explicit labels from the task, could support a label-less, pure-exemplar account. However, just because we did not introduce labels does not mean that participants didn’t activate something label-like in the experiment, consciously or subconsciously.

While we have shown this effect based on words and objects with real, long-term associations with gendered speakers, we might predict that these indexical generalization effects would hold in lab-introduced associations as well. For example, if we exposed participants to new vocabulary that they heard primarily from male or female speakers, we predict that they would then associate these words with male and female objects. Conversely, if we introduced participants to novel objects, contextualising them as gendered, we predict that participants would show an association between these objects and gendered words, even though they have never heard them together.

### Methodological implications

Our results and methodology have implications for the wider IAT-using community. In terms of our results, in experiments 1, 2 and 3, we replicate Rothermund & Wentura [53] by showing that participants are faster when REAL-WORD and YOUNG are paired, and similarly, when REAL-WORD and a participant’s own gender are paired. This provides support for Rothermund & Wentura’s argument that the shared markedness/salience of paired categories may be as important as shared semantics.

Methodologically, as pointed out by de Houwer [74], there is normally a confound between category- and member-valence in the classic IAT design: it is hard to say whether it is the category label FLOWER or the category members like *tulip* that are positively valenced, though de Houwer’s attempt at separating this confound suggests that the valence of the category label is what matters in standard IAT designs (see also [75, 76]). Other researchers, however, have argued that stimuli items do matter [77–79]. All of these studies apart from de Houwer have used a cross-block/participants design. In our study, both category-congruent and -incongruent words were sorted with the same hand at the same time, as the sorting task for target items was a lexical decision task, and both types of words were real words. Since we find differences between the real words when they are paired with different gendered labels, we are able to say that the word-gender effect is about more than an association/facilitation for REAL WORD and for example, the category label FEMALE. Rather, it is a subset of those real words—the ones that participants have experienced more often coming from women—that are accessed faster in the FEMALE-REAL WORD pairing. Researchers, even those not interested in language specifically, might find the combined IAT-lexical decision task useful in distinguishing between associations based on category label, and associations with category members.

Relatedly, in experiment 4 we saw our critical effect emerge even with the use of irrelevant labels. This is not to say that labels didn’t matter; rather, as a block progressed participants learnt that frames were consistently around FEMALE/MALE objects, despite explicit labels not being introduced. This demonstrates that participants can invoke and use category labels even when they are not explicitly mentioned in a task [80], and could potentially be applied to understanding what sort of labels participants generate on their own, especially in cases where it is unclear what “nominal features” are activated by certain categories [56] (p. 426–427).

## Conclusion

In this study we presented the results of four combined Lexical Decision and IAT experiments, which all investigated whether lexical access was facilitated when words that participants have experienced more from certain types of speakers were paired with faces of people in that social group, or with objects stereotypically associated with the social group. We find evidence to support this hypothesis in all experiments. Critically, the results suggest that people’s socially-skewed experiences with words result in not just an association between the word and certain types of speakers, but with the word and more abstract social categories. We argue that this provides further support for hybrid models of lexical representation, in which both linguistic and social categories are associated with phonetically detailed lexical storage.

## Supporting information

S1 Appendix Real and nonsense words used in experiment 1. Due to experimental error, the non-words gid/gozz varied across participants.
(DOCX)

S2 Appendix Real and nonsense words used in experiments 2, 3, and 4. Due to experimental error, the non-word chilkres was presented as chilkes for experiment 3.
(DOCX)
