# Supplementary material for: Abstract social categories facilitate access to socially skewed words
Source: PLoS One. 2019 Feb 4;14(2):e0210793. doi: 10.1371/journal.pone.0210793 (PMC6361498; doi:10.1371/journal.pone.0210793)
Supplement: S1 Appendix — Due to experimental error, the non-words gid/gozz varied across participants. (DOCX) [file pone.0210793.s001.docx]

S1. *Appendix. Real and nonsense words used in experiment 1. Due to experimental error, the non-words gid/gozz varied across participants.*

| **old** | **young** | **nonwords** | |
| --- | --- | --- | --- |
| beg | bitten | bantils | dar |
| electricity | environment | faleworgs | fifeo |
| hymns | impressive | frife | gid/gozz |
| mittens | nicest | homns | infelleptual |
| respected | spirits | laperies | meddons |
| confectionery | chemistry | moners | oble |
| fireworks | expensive | onpironment | rifeshile |
| idle | intellectual | sorent | straff |
| pencils | physics | teg | vasanth |
| silent | stress | vifficks | wippor |
| delivered | depressing | chalmestry | axfonsive |
| fried | fridge | febivered | defreving |
| libraries | lifestyle | zerected | freddon |
| permission | reckon | impreshive | heckam |
| whistle | survival | locite | kishest |
| dental | dye | nidge | mofting |
| frighten | guide | pretessiom | oloflippity |
| miners | lifting | spalats | simimal |
| recite | seventh | tomlectionary | tansal |
| willow | video | washle | vibben |
