# Supplementary material for: Abstract social categories facilitate access to socially skewed words
Source: PLoS One. 2019 Feb 4;14(2):e0210793. doi: 10.1371/journal.pone.0210793 (PMC6361498; doi:10.1371/journal.pone.0210793)
Supplement: S2 Appendix — Due to experimental error, the non-word chilkres was presented as chilkes for experiment 3. (DOCX) [file pone.0210793.s002.docx]

*S2. Appendix. Real and nonsense words used in experiments 2, 3, and 4. Due to experimental error, the non-word chilkres was presented as chilkes for experiment 3.*

| **Female** |  | **Male** |  | **Non-Words** | |  |  |
| --- | --- | --- | --- | --- | --- | --- | --- |
| patients | | fifty |  | affiftank | | fett |  |
| parents | | company | | approticegoft | | fickorshall | |
| ward |  | church | | bainy |  | fulp |  |
| homework | | bands |  | behimine | | gaffminy | |
| happy |  | cameras | | beshints | | glarm |  |
| assistant | | vehicle | | boofic | | gooze |  |
| dad |  | guard |  | carmenters | | gormers | |
| shells | | licence | | ceifar | | gruss |  |
| netball | | corners | | chilkres | | heabmaller | |
| sewing | | net |  | churk |  | himehock | |
| library | | apprenticeship | | clant |  | igent |  |
| lovely | | bunch |  | colber | | lafimp | |
| headmaster | | guitar | | colfanies | | miffs |  |
| teacher | | companies | | cozzy |  | naing |  |
| children | | engineering | | cratt |  | nosfelt | |
| mum |  | plant |  | cume |  | onkay |  |
| buses |  | financial | | cutibom | | paifess | |
| cute |  | nerves | | dode |  | pisnoff | |
| pony |  | trout |  | dotcher | | plofe |  |
| auntie | | music |  | dreap |  | poot |  |
| treat |  | business | | enginaining | | rortory | |
| mother | | agent |  | fabiter | | ruvler | |
| uniform | | bloke |  | falents | | sarking | |
| girls |  | capital | | favdy |  | sheebs | |
| class |  | guys |  | veinds | | trouge | |
